# Parameter optimization and mechanism of action of Light‑Emitting Diode phototherapy in a rat model of bacterial vaginosis

**DOI:** 10.3389/fcimb.2026.1836549

**Published:** 2026-10-01

**Authors:** Xiuxin Chen, Xinyi Qi, Yuan Ma, Bowei Li, Yu‑e Yang, Zhuo Xiong, Yanhui Yang, Chunfang Ha

**Affiliations:** 1 Ningxia Medical University, Yinchuan, China; 2 Department of Gynecology, General Hospital of Ningxia Medical University, Yinchuan, China; 3 Ningxia Key Laboratory of Infection and Immunity, School of Basic Medical Sciences, Ningxia Medical University, Yinchuan, China; 4 Key Laboratory of Fertility Preservation and Maintenance of Ministry of Education, Ningxia Medical University, Yinchuan, China

**Keywords:** bacterial vaginitis, bactericidal mechanism, *Escherichia coli*, *Gardnerella vaginalis*, Light‑Emitting Diode

## Abstract

**Objective:**

To investigate the antibacterial effects of LED phototherapy on *Gardnerella vaginalis* (*Gv*) and *Escherichia coli* (*E. coli*), optimize key irradiation conditions for LED phototherapy by establishing an *in vivo* infection model, and evaluate its feasibility for treating bacterial vaginosis.

**Methods:**

Bacterial colonies were irradiated with LEDs at different wavelengths (405 nm, 415 nm, 430 nm, 630 nm, 850 nm). Bacterial survival and morphology were assessed via colony counting, SYTO9/PI fluorescence staining, and scanning electron microscopy. Intracellular oxidative stress and antioxidant systems were quantitatively measured. Genomic DNA integrity was analyzed by agarose gel electrophoresis to validate the bactericidal mechanism. The effects on vaginal epithelial cell (VK2/E6E7). viability were assessed using CCK-8 assays, ROS detection, and SYTO9/PI live/dead staining. A rat model of bacterial vaginosis was established to optimize vaginal LED irradiation parameters, with inflammatory cell infiltration assessed by H&E staining, and expression levels of IL-1β and TNF-α in vaginal tissues evaluated by immunohistochemistry (IHC) with semi-quantitative image analysis.

**Results:**

Among the tested wavelengths, 415 nm LED demonstrated optimal antibacterial efficacy. At 50 mW/cm² intensity for 30 min, it exhibited the most significant bactericidal effect against *Gv* and *E. coli*. This condition induced substantial ROS production in bacteria, inhibited SOD and CAT activity, leading to bacterial cell membrane damage and genomic DNA fragmentation. This irradiation condition exhibited no significant toxicity to vaginal epithelial cells VK2/E6E7. In a rat model, LED treatment under these conditions significantly improved vaginal inflammation symptoms and pathological histological findings, while reducing IL- 1β and TNF-α expression.

**Conclusion:**

This study established the optimal LED irradiation conditions for BV-associated pathogens as 415 nm wavelength, 50 mW/cm² power, and 30 min exposure (irradiation dose 90 J/cm²). These conditions exert significant antibacterial effects against *Gv* and *E. coli* by inducing bacterial ROS production, while maintaining excellent cellular safety. In rat cell models, it simultaneously demonstrated anti-inflammatory and tissue repair potential in both bacterial and *in vivo* settings, offering a novel therapeutic strategy for the clinical management of bacterial vaginosis.

## 1 Introduction

The dynamic microbial balance of the vaginal microbiome is crucial for maintaining vaginal physiological function (Chen et al., 2021; El-Sayed et al., 2021). Disruption of this balance can easily trigger various types of vaginal inflammation. Bacterial vaginosis (BV) is the most common form of vaginitis, Global epidemiological studies indicate a prevalence rate of approximately 23%–29% (Swidsinski et al., 2023). It primarily results from an imbalance in the vaginal microbiome, characterized by a decrease in Lactobacillus species and overgrowth of *Gv*. Its clinical manifestations include increased vaginal discharge with a “fishy odor,” and some patients may experience vaginal or vulvar itching (Majigo et al., 2021). Existing research indicates that BV is closely associated with multiple adverse obstetric and gynecological outcomes, including preterm birth (Klebanoff et al., 2023; Steetskamp et al., 2024), miscarriage, pelvic inflammatory disease (Ravel et al., 2021), and increased risk of human immunodeficiency virus (HIV) infection (Armstrong and Kaul, 2021). Therefore, BV not only impacts patients’ quality of life but also poses a serious threat to their physical and mental health.

Current first-line treatment for BV primarily relies on antibiotics, with metronidazole and clindamycin being the most commonly used drugs. However, factors such as bacterial evolution and genetic mutations have led to increased bacterial resistance or insufficient bactericidal effects of antibiotics, resulting in diminished therapeutic efficacy. The recurrence rate of BV within six months after metronidazole treatment reaches as high as 50% (Braunstein and Selk, 2024), and long-term use carries the risk of developing antibiotic resistance (Tomás et al., 2020). Therefore, exploring a new, safe, and effective treatment approach is particularly important.

Light-emitting diodes (LEDs) are electronic devices that can emit light of different wavelengths. Blue light within this spectrum is emerging as a novel antimicrobial approach, demonstrating significant bactericidal potential against multiple bacterial strains in recent years (Arifianto et al., 2025; Gonçalves et al., 2025). This technology leverages specific wavelengths of blue light to activate endogenous photosensitizers (400–500 nm) within bacteria, triggering photodynamic reactions that generate reactive oxygen species to selectively eliminate pathogens. Research confirms that numerous bacteria synthesize porphyrins (Hamblin et al., 2005; Kumar et al., 2015),though their types and concentrations exhibit species specificity (Plavskii et al., 2018).Although antibacterial blue light demonstrates promising bactericidal effects *in vitro*, systematic research on LED irradiation conditions for BV treatment remains lacking. Key parameters—including optimal antibacterial wavelengths, irradiation power, and exposure duration—remain undefined. Furthermore, the antibacterial mechanisms under different light conditions, cellular safety, and optimization of *in vivo* irradiation parameters constitute core challenges hindering this technology’s clinical translation for BV. Meanwhile, the relationship between LED phototherapy conditions and vaginal microecological preservation or local anti-inflammatory effects in BV treatment remains unvalidated.

This study aims to establish a rat vaginitis model to systematically investigate the antibacterial effects of light-emitting diode (LED) light sources against *Gv* and *E. coli*. Through *in vitro* experiments, we will identify the optimal antibacterial wavelength, determine the best irradiation power and duration at this wavelength, and evaluate the feasibility and safety of this technology for treating vaginitis. This research seeks to provide novel therapeutic approaches and theoretical foundations for clinical practice.

## 2 Methods

### 2.1 *Gv* and *E. coli* strains and culture conditions

*Gv* was purchased from (Beina Bio BNCC354890). The lyophilized *Gv* powder was resuspended in PBS and inoculated onto Columbia blood agar solid medium. The plate was then placed in a sealed bag containing an anaerobic gas-producing bag and incubated at 37 °C for 24–48 hours. Successful activation was indicated by the formation of a circular white colony at the needle tip. The colony was then picked and added to brain heart infusion broth(BHI) containing yeast, cultured anaerobically at 37 °C at 220 rpm for 24–48 hours. *E. coli* was purchased from (Mingzhou Bio ATCC25922). *E. coli* was similarly inoculated onto BHI agar and cultured at 37 °C for 12 hours. Upon successful bacterial activation, a single colony is picked and cultured in BHI at 37 °C for 12 hours. The two bacteria have different cultivation conditions.

### 2.2 Light source

Irradiation with red and blue light-emitting diodes (LEDs) ofdifferent wavelengths, at an irradiance of 50 mW/cm². The LED light panel was developed by Beijing Chuangying Technology Medical Co., Ltd.

### 2.3 Bactericidal activity assay

Two bacterial strains were inoculated into 10 mL of liquid medium and cultured. Upon reaching the mid-logarithmic phase, the bacterial suspensions were harvested, centrifuged at 5,000×g for 10 min, and washed twice with phosphate-buffered saline (PBS). The initial optical density at 600 nm (OD_600_) was adjusted to approximately 0.7. Aliquots of 300 µL of the bacterial suspension were dispensed into each well of a 96-well plate. The plates were then irradiated for 50 min under an LED array emitting at wavelengths of 405, 415, 430, 630, and 850 nm, each at a power density of 50 mW/cm². Control groups were kept in complete darkness throughout the experiment. To preliminarily evaluate the bactericidal efficacy of different wavelengths, the OD_600_ of each well was measured every 10 min using a microplate reader, and the resulting survival curves were monitored for a declining trend following LED exposure.

### 2.4 Colony-forming unit assay

After each illumination cycle, perform serial dilutions of *Gv* and *E. coli* samples at specified dilution factors. Take 100 μL of Gv suspension and spread onto Columbia blood agar plates. Incubate in anaerobic gas-producing bags at 37 °C for 24 hours to determine colony-forming units per milliliter (CFU/mL). Take 100 μL of*E. coli* suspension and spread evenly onto BHIA plates. Incubate at 37 °C for 12 hours to determine CFU/mL.

### 2.5 Bacterial dead/live detection

Employing a dual-staining method with SYTO9 (Thermo Fisher Scientific, USA) and propidium iodide (PI, Lab Lead, Beijing), the viability status of *Gv* and *E. coli* was observed under a fluorescence microscope. After LED illumination, bacterial suspensions were collected by centrifugation at 5000×g for 10 min to pellet cells. Cells were then stained in the dark with a SYTO9/PI mixed solution for 15 min. Following staining, samples were washed twice with PBS and resuspended in PBS. A 10 μL drop of the bacterial suspension was placed on a microscope slide and observed under a fluorescence microscope (Olympus, Japan). SY red fluorescence. Finally, quantitative analysis of the fluorescence images was performed using ImageJ software.

### 2.6 Scanning electron microscopy

After treating *Gv* and *E. coli* bacterial suspensions with 415 nm LED illumination (irradiance 50 mW/cm²) for 30 minutes, centrifuge at 5000×g for 10 minutes and collect 1 mL of bacterial cells from each. Fix the samples using electron microscopy fixative, then apply gold coating to enhance conductivity.

Finally, observe morphological changes in both bacterial species using scanning electron microscopy.

### 2.7 Cell membrane permeability assay

Two bacterial suspensions were subjected to light exposure (under the same conditions as for scanning electron microscopy observation), followed by centrifugation to collect the cells. Since nucleic acids and proteins exhibit characteristic absorption peaks at 260 nm and 280 nm, respectively, changes in absorbance at these wavelengths can reflect alterations in cell membrane permeability. Using a microplate reader (Thermo Fisher Scientific, USA), absorbance values at 260 nm and 280 nm were measured to assess bacterial cell membrane integrity and permeability status.

### 2.8 Detection of bacterial ROS content

DCFH fluorescent probes (SolaBio Biotechnology Co., Ltd., Beijing) were used to measure endogenous reactive oxygen species (ROS) levels in bacteria. This probe penetrates bacterial cell membranes and reacts with intracellular ROS to generate green fluorescence, with fluorescence intensity positively correlated to ROS levels. After LED exposure, bacterial cells were collected by centrifugation at 5000×g for 5 min. Cells were resuspended in PBS containing 10 μM DCFH probe and incubated at 37 °C in the dark for 15 min. Subsequently, cells were washed twice with PBS to remove extracellular residual probe and reduce background interference. After resuspending the cells, 200 μL of bacterial suspension was added to each well of a 96-well plate. Detection was performed using a fluorescence microplate reader. The excitation wavelength was set to 497 nm, and the emission wavelength was set to 525 nm. The fluorescence signal intensity was measured to quantitatively analyze the intracellular ROS production levels in bacteria.

### 2.9 Detection of bacterial endogenous antioxidant enzyme activity

Superoxide Dismutase (SOD) and Catalase (CAT) are key antioxidant enzymes widely present in various cells and bacteria. Analysis of the *Gv* genome sequence in the NCBI database revealed that the currently known *Gv* genome does not contain gene sequences encoding SOD and CAT. Therefore, this study focused solely on determining antioxidant enzyme activity in *E. coli*. *E. coli* subjected to LED intervention was centrifuged at 5000×g for 10 min to collect bacterial pellets. After resuspension in 1 mL PBS, the pellets were placed in an ice bath and disrupted using an ultrasonic homogenizer to release intracellular enzymes into the lysate supernatant. The supernatant was collected by centrifugation at 4 °C. Subsequently, the enzyme activity levels were quantitatively determined according to the protocols provided in the SOD and CAT activity assay kits, respectively.

### 2.10 Bacterial DNA damage

The extent of bacterial DNA damage following LED exposure was analyzed via agarose gel electrophoresis. Suspensions of *Gv* and *E. coli* exposed to LED light were preliminarily collected, centrifuged to obtain bacterial pellets, and genomic DNA extracted from both strains using bacterial genomic DNA extraction kits (Nanjing Jiancheng Company). The resulting DNA samples were loaded into 1% agarose gel wells for electrophoretic separation. Following electrophoresis, DNA band morphology was observed and recorded under an UV imaging system for qualitative assessment of DNA damage.

### 2.11 Immortalized vaginal epithelial cell culture

Utilize the immortalized human vaginal epithelial cell line VK2/E6E7 (deposited at ATCC, USA). VK2/E6E7 cells are cultured in Keratinocyte-Serum Free Medium (K-SFM, Gibco). Incubate at 37 °C with 5% CO_2_ for 48–72 hours. When cells reached 80% confluence, they were digested with trypsin and passaged at a 1:2 ratio.

### 2.12 CCK8 assay

Seed VK2/E6E7 cells at a density of 1×10^5^ cells per well in a 96-well plate. Incubate at 37 °C with 5% CO_2_ for 24 hours to allow cells to adhere.

Once cells were fully adherent, remove the original medium and add 100 μL of PBS buffer to each well. Place the 96-well plate under an LED light plate (415 nm, 50 mW/cm²) for irradiation. Ensure adequate heat dissipation during irradiation to prevent thermal effects from interfering with cell viability. After irradiation, remove the PBS buffer and replace each well with 100 μL D-KSFM medium. Continue incubation in the cell culture incubator for 4 hours. Subsequently, add 10 μL of CCK-8 solution (Biyuntian Biotechnology) to each well. Incubate in the incubator under dark conditions for 2 hours. Measure the absorbance at 450 nm using a microplate reader to assess cellular metabolic activity and proliferation status.

### 2.13 ROS detection in VK2/E6E7 cells

Cell seeding, culture, and LED irradiation procedures were performed as described previously. Upon completion of cell treatment, immediately remove the PBS buffer from the wells. Add 50 μL of PBS solution containing 10 μM DCFH-DA probe to each well. Incubate the plate in a 37 °C dark incubator for 15 minutes. After incubation, gently wash the cells twice with PBS to remove unincorporated probe. Finally, detect using a fluorescence microplate reader with an excitation wavelength of 488 nm and an emission wavelength of 525 nm. Quantify intracellular ROS levels by recording fluorescence intensity.

### 2.14 Cell live/dead staining

After completing the intervention treatment, add the prepared Live/Dead dual-stain working solution to each well. Subsequently, place the culture plate in a 37 °C constant-temperature incubator for 15 minutes of dark incubation. Following incubation, gently wash the cells twice with pre-warmed PBS buffer to remove excess staining reagent. Finally, observe and capture images of the samples under an inverted fluorescence microscope.

### 2.15 Rat animal model establishment

Female SD rats aged 6–7 weeks (purchased from Shanghai SLAKE Laboratory Animal Co., Ltd.) were used in this study and housed at the Laboratory Animal Center of Shanghai Chengxi Biotechnology Co., Ltd. Housing conditions included a 12-hour light-dark cycle, ambient temperature of 20–26 °C, relative humidity of 50%–60%, and free access to food and water. All animal experimental protocols were approved by the Animal Ethics Committee of Shanghai Chengxi Biotechnology Co., Ltd. (Approval No.: CX052411044). After 7 days of acclimation, rats received subcutaneous injections of estradiol benzoate (1 mg/kg) for 3 consecutive days to establish an estrogen-dominant vaginal microenvironment. Subsequently, a mixed bacterial suspension containing *Gv* and *E. coli* (bacterial ratio 10:1 (Shang et al., 2024), prepared according to reference methods) was administered via vaginal instillation for 7 consecutive days. Vaginal microbiota status was assessed using the Nugent scoring system, with a score ≥7 indicating successful establishment of the bacterial vaginosis model. Successfully modeled rats were randomly divided into four groups (n=4 per group): Normal Control (NC), Model Control (AC, no specific intervention), LED Treatment (LED), and Metronidazole Suppository Treatment (Me). Intervention protocols for each group were as follows: The LED group received intravaginal irradiation with 415 nm LED light, while the Me group received daily intravaginal administration of metronidazole suppositories. Both interventions were administered continuously for 7 days.

### 2.16 Rat vaginal LED irradiation system

The LED irradiation equipment used in this study was provided by Beijing Chuangying Medical Technology Co., Ltd. Prior to irradiation, rats were anesthetized with inhaled isoflurane and secured to the operating table. The fiber optic probe was gently inserted approximately 1 cm into the vagina to ensure precise targeting of the irradiation area. Vaginal washout fluid samples were collected on the intervention start date (Day 0) and on Day 5 of the intervention for subsequent 16S rRNA gene sequencing analysis.

### 2.17 Hematoxylin and eosin staining

Following intervention completion, rats were euthanized by cervical dislocation, and vaginal tissues were dissected. Tissue samples were fixed in 4% paraformaldehyde, paraffin-embedded, sectioned, and subjected to hematoxylin-eosin (HE) staining. Histopathological changes, including vaginal mucosal structure, epithelial integrity, and inflammatory cell infiltration, were examined under a light microscope. HE staining was used for morphological assessment only.

### 2.18 Immunohistochemical detection

IHC was employed to detect and semi-quantitatively evaluate the expression levels of inflammatory cytokines IL-1β and TNF-α in vaginal tissues. Primary antibody dilutions were as follows: rabbit anti-IL-1β monoclonal antibody (1:150), rabbit anti-TNF-α monoclonal antibody (1:500). Paraffin sections underwent dewaxing, antigen retrieval, and serum blocking. The corresponding primary antibodies were applied and incubated overnight at 4 °C. Staining was performed using HRP-labeled secondary antibodies and a DAB chromogenic system. For quantitative analysis, five randomly selected high-power fields (×400) per section were captured, and the integrated optical density (IOD) of positive staining was measured using ImageJ software.

### 2.19 Statistical methods

Statistical analysis was performed using GraphPad Prism 9.0 software. All experiments were independently repeated three times. A p-value < 0.05 was considered statistically significant.

## 3 Result

### 3.1 Effect of LEDs on bacterial counts

The bactericidal efficacy of LEDs at different wavelengths against *Gv*is shown in **Figure 1A**. In *Gv*, all groups exhibited a decreasing trend in OD600 values. However, the 415 nm wavelength LED caused the most significant decrease in OD600, which stabilized after 30 minutes. In *E. coli* (**Figure 1B**), the control group showed no significant change in OD600 values. After 10 minutes of exposure to 405 nm wavelength LEDs, OD600 values began to decrease. Exposure to 415 nm wavelength LEDs for 10 minutes resulted in a significant decrease in OD600 values. But the OD600 value decreased after 50 minutes of exposure to 430 nm wavelength LEDs.

**Figure 1:**
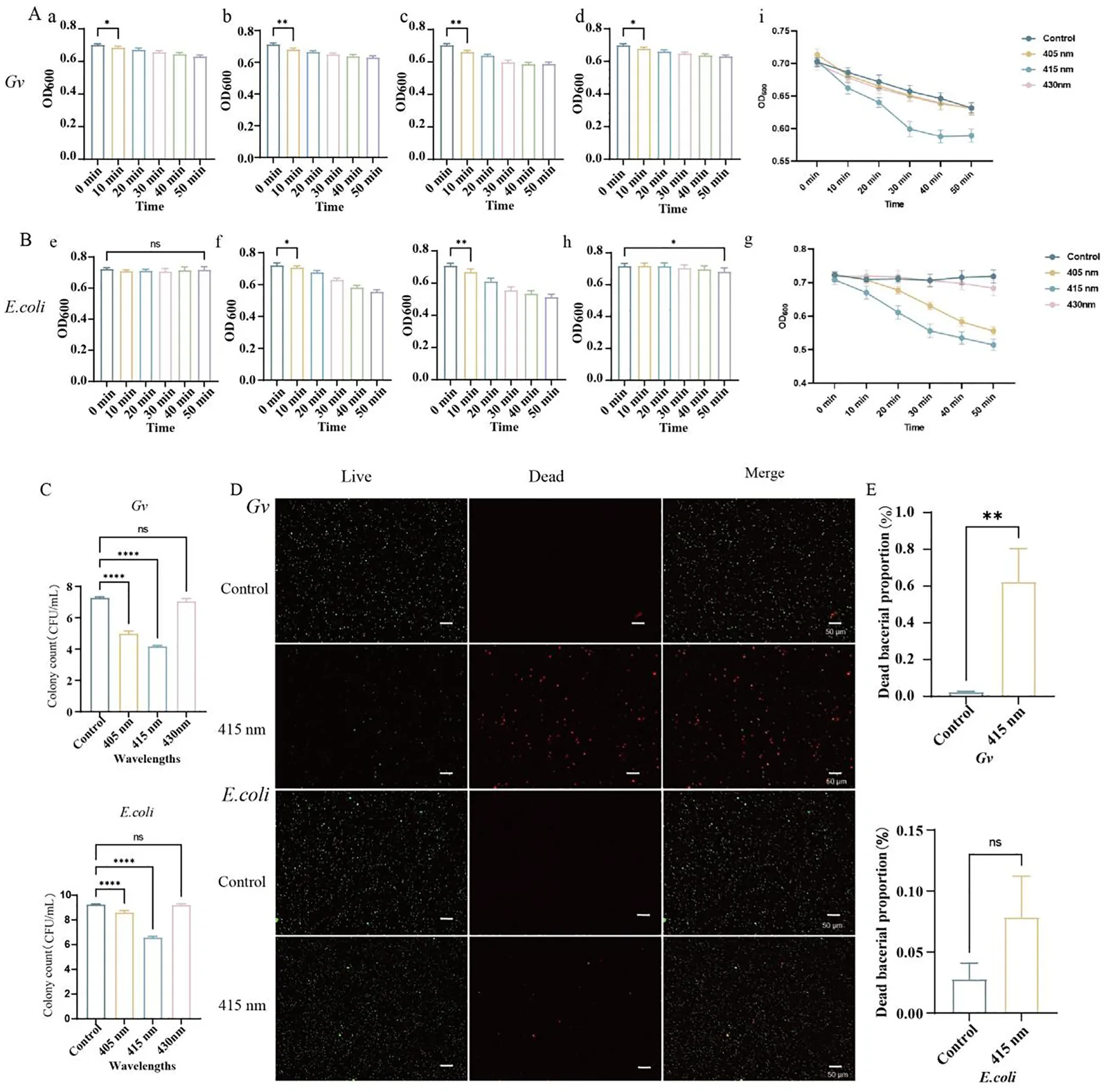
Effects of LED on *Gv* and *E. coli* quantity. **(A)** Effects of LEDs in different groups on *Gv* OD600 values at different times. **(a)** Control, b: 405 nm, **(c)** 415 nm, **(d)** 430 nm; **(B)** Effects of LEDs in different groups on *E. coli* OD600 values at different times. **(e)** Control, **(f)** 405 nm, **(g)** 415 nm, **(h)** 430 nm; **(C)** Colony counts after 30 min of LED irradiation in different groups; **(D)** Live/Dead staining for observing *Gv* and *E. coli*, live bacteria show green fluorescence and dead bacteria show red fluorescence; **(E)** Statistical chart of the percentage of dead bacteria. *P < 0.05, **P<0.01, ****P < 0.0001.

Similar to *Gv*, *E. coli* exhibited the most pronounced decrease in OD600 value after exposure to 415 nm wavelength LEDs, with both bacterial strains stabilizing after 30 minutes of illumination. Therefore, the effect of 30-minute exposure to LEDs of various wavelengths on the colony counts of both bacterial strains was further validated. As shown in **Figure 1C**, compared with the control group (7.277 ± 0.054 log_10_ CFU/mL), the viable count of *Gv* was significantly reduced to 4.989 ± 0.137 log_10_ CFU/mL upon irradiation at 405 nm, and further decreased to 4.181 ± 0.052 log_10_ CFU/mL at 415 nm. In contrast, irradiation at 430 nm resulted in no notable change in bacterial counts (7.055 ± 0.165 log_10_ CFU/mL) relative to the control.log10 CFU/mL. For *E. coli*, compared with the control group (9.2596 ± 0.0404 log_10_ CFU/mL), the viable count decreased to 8.6159 ± 0.1362 log_10_ CFU/mL following 405 nm irradiation, and further dropped to 6.5834 ± 0.0955 log_10_ CFU/mL upon 415 nm irradiation. In contrast, no significant reduction was observed after 430 nm LED exposure, with the count remaining at 9.2155 ± 0.0982 log_10_ CFU/mL. Among the tested wavelengths, 415 nm LED irradiation for 30 min exhibited the most potent bactericidal effect against both *Gv* and *E. coli*.

Live/Dead bacterial staining results showed that, compared with the control group, the number of dead *Gv* and *E. coli* bacteria significantly increased, while the number of viable bacteria markedly decreased after irradiation with 415 nm LED light (**Figures 1D, E**). Therefore, 415 nm wavelength LED was adopted in subsequent experiments to explore its bactericidal mechanisms against *E. coli* and *Gv*.

### 3.2 LED sterilization mechanism

To thoroughly investigate the mechanism of LED light’s bactericidal effect on *Gv* and *E. coli*, this study conducted a systematic analysis across multiple dimensions, including bacterial morphology, ROS levels, nucleic acid and protein macromolecular leakage, and DNA damage. Scanning electron microscopy revealed that *Gv* cells in the control group exhibited intact morphology with smooth cell membranes and no apparent structural damage. In contrast, after exposure to 415 nm LED light (50 mW/cm², 30 min), *Gv* cells displayed ruptured cell membranes, cytoplasmic leakage, and fragmentation (**Figure 2A**). Similar alterations were observed in *E. coli*: control group cells maintained intact structures, whereas LED-treated group exhibited significant membrane disruption accompanied by cell fragmentation. Notably, under identical illumination conditions, *Gv* cells sustained more pronounced structural damage than *E. coli*.

**Figure 2:**
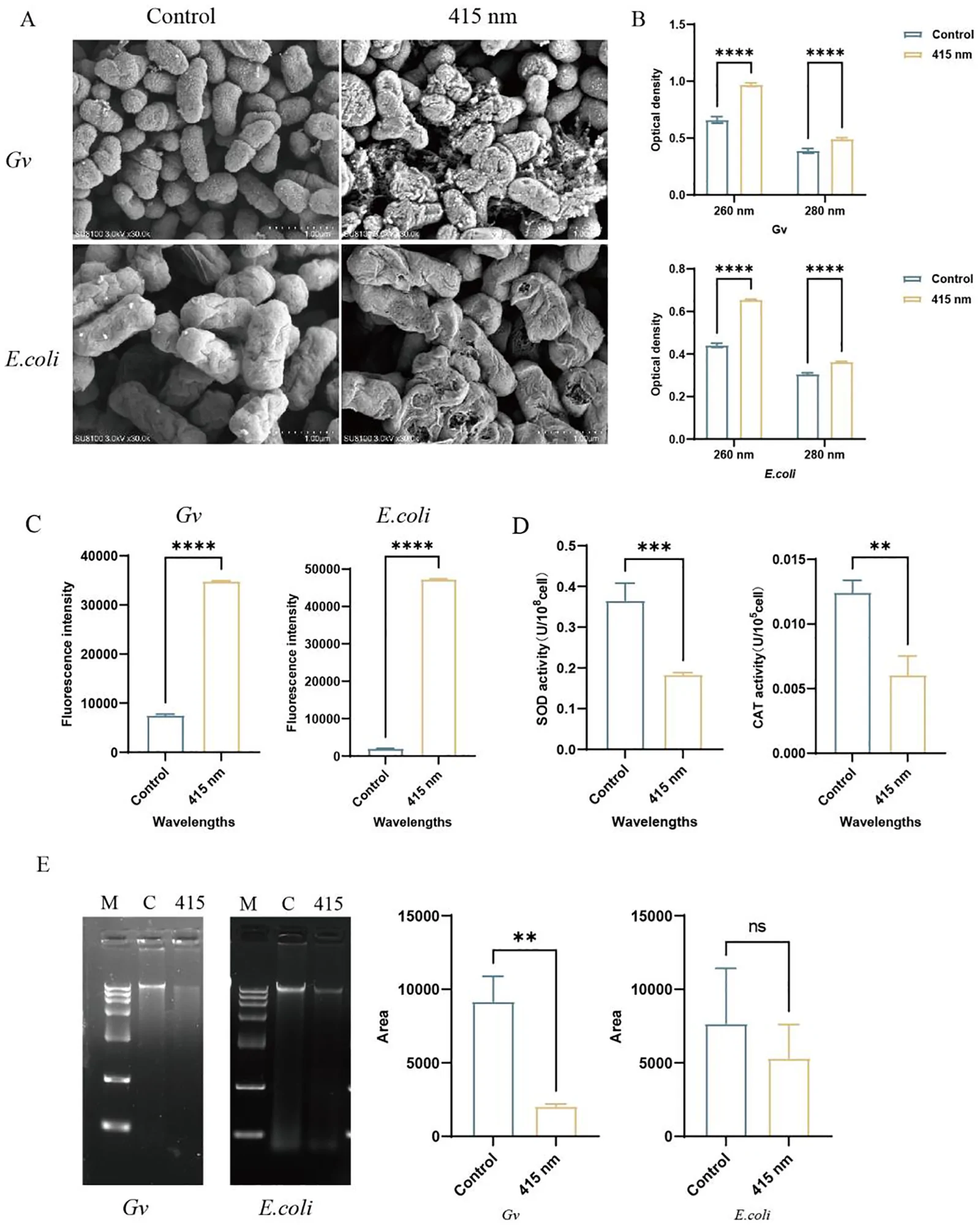
Effect of LEDs on *Gv* and *E. coli*. **(A)** *Gv* and *E. coli* SEM images (magnification 30 K×); Absorbance values of **(B)** *Gv*, *E. coli* supernatants at 260 nm and 280 nm; **(C)** *Gv* and *E. coli* ROS contents; **(D)** *E. coli* SOD and CAT activity assays; **(E)** *Gv* and *E. coli* bacterial genomic DNA agarose gel electrophoresis and band quantification **P<0.01, ***P<0.001, ****P<0.0001.

As a key structure maintaining intracellular environmental stability, the cell membrane possesses selective permeability, normally preventing the leakage of macromolecules such as nucleic acids and proteins. This study evaluated the impact of LED illumination on cell membrane permeability using a dual-staining method with SYTO9/PI combined with absorbance measurements of nucleic acids (OD260) and proteins (OD280) in the supernatant. The results showed that the absorbance values of the culture supernatants from both *G. vaginalis* and *E. coli* groups at 260 nm and 280 nm were significantly increased following LED irradiation, as compared with the respective control groups (**Figure 2B**). This indicates damage to the bacterial membrane structure, increased permeability, and the leakage of intracellular macromolecules.Following exposure to 415 nm LED irradiation, substantial ROS generation was observed in both *Gv* and *E. coli*. Results indicated that ROS levels were low in the control group of *Gv*(7503.7 ± 259.2 a.u), while ROS content significantly increased in the LED-irradiated Group(34862.7 ± 59.5 a.u).A similar phenomenon was observed in *E. coli*, where intracellular ROScontent was extremely low in the control group(2024.3 ± 27.5 a.u), whereas ROS levels markedly elevated in the LED-treated group(47267.0 ± 112.0 a.u) (**Figure 2C**).

Within microorganisms, oxidation and antioxidation typically maintain a dynamic equilibrium. To investigate bacterial antioxidant capacity, this study further measured the activity of key intracellular antioxidant enzymes—SOD and CAT. Results showed that in *E. coli*, both SOD and CAT activities were significantly reduced after LED irradiation. (**Figure 2D**) Since no gene sequences encoding SOD or CAT were identified in the current *Gv* genome data, enzyme activity assays were not performed for *Gv*. Finally, to evaluate the DNA-damaging effects of LEDs on bacteria, agarose gel electrophoresis analysis was performed on genomic DNA from both bacterial species. Results indicated that cumulative LED irradiation doses of 90 J/cm² at 415 nm caused significant degradation and damage to the genomic DNA of both *Gv* and *E. coli* (**Figure 2E**).

### 3.3 Exploration of LED light conditions on the immortalized vaginal epithelial cell line VK2/E6E7

Based on previous bacterial experiments showing that 415 nm LED irradiation exhibited optimal antibacterial efficacy, this study evaluated the biosafety of this wavelength in VK2/E6E7 cells and its potential effects on host cells. To investigate the effect of LED irradiation on cell proliferation, the CCK-8 assay was used to measure cellular metabolic activity after different irradiation durations **Figure 3A**). The results showed that VK2/E6E7 cell viability gradually increased with prolonged irradiation. Under the same LED irradiation conditions, only a small amount of reactive oxygen species (ROS) was detected in VK2/E6E7 cells compared with the control group (**Figure 3B**). In addition, Live/Dead dual staining showed no significant increase in VK2/E6E7 cell death after 30 min of LED irradiation compared with the non-irradiated group (**Figure 3C, D**). These results suggest that LED irradiation at a wavelength of 415 nm and a light power density of 50 mW/cm^2^ can effectively inhibit bacterial growth without inducing obvious cytotoxicity in VK2/E6E7 cells.

**Figure 3:**
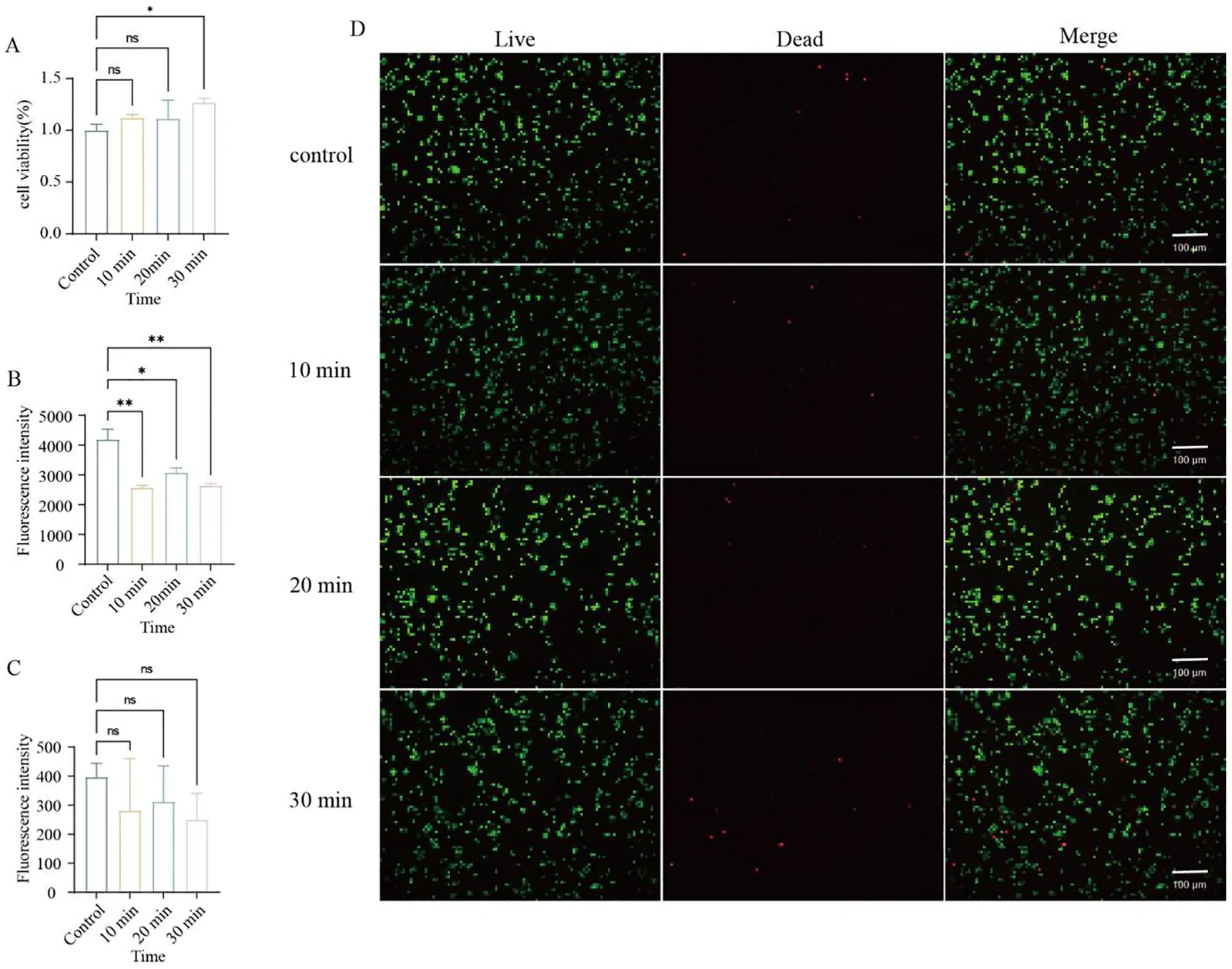
Effect of LED on the activity of VK2/E6E7 cells. **(A)** CCK8 detected the metabolic activity of cells; **(B)** ROS content detection; **(C)** Live/Dead staining, red fluorescence statistical chart; **(D)** VK2/E6E7 cells were stained with Live/Dead staining *P<0.05, **P<0.01.

### 3.4 Exploration of optimal treatment conditions for LED therapy in rat models

This study systematically evaluated the structural characteristics of vaginal microbiota across different treatment groups by calculating microbial community α-diversity indices, including the CHAO1 index, Shannon index, and Simpson index. CHAO1 index analysis revealed no significant difference in microbial richness between the model group (AC) and the normal control group (NC) (P>0.05). However, statistically significant differences were observed between the model group (AC) and both the LED treatment group (LED) and the metronidazole treatment group (Me)(P<0.05), indicating that intervention treatments significantly influenced community species richness (**Figure 4B**). Shannon index analysis further revealed trends in species richness and community evenness across groups (**Figure 4C**). Simpson index results showed no significant differences between the model group and other groups (**Figure 4D**) (P>0.05), indicating relatively stable dominant species composition across groups. Further analysis revealed that after LED light exposure and metronidazole treatment, the abundance of rare species in the vaginal microbiota decreased, while the abundance of dominant species remained unchanged, and species distribution tended toward greater uniformity.

**Figure 4:**
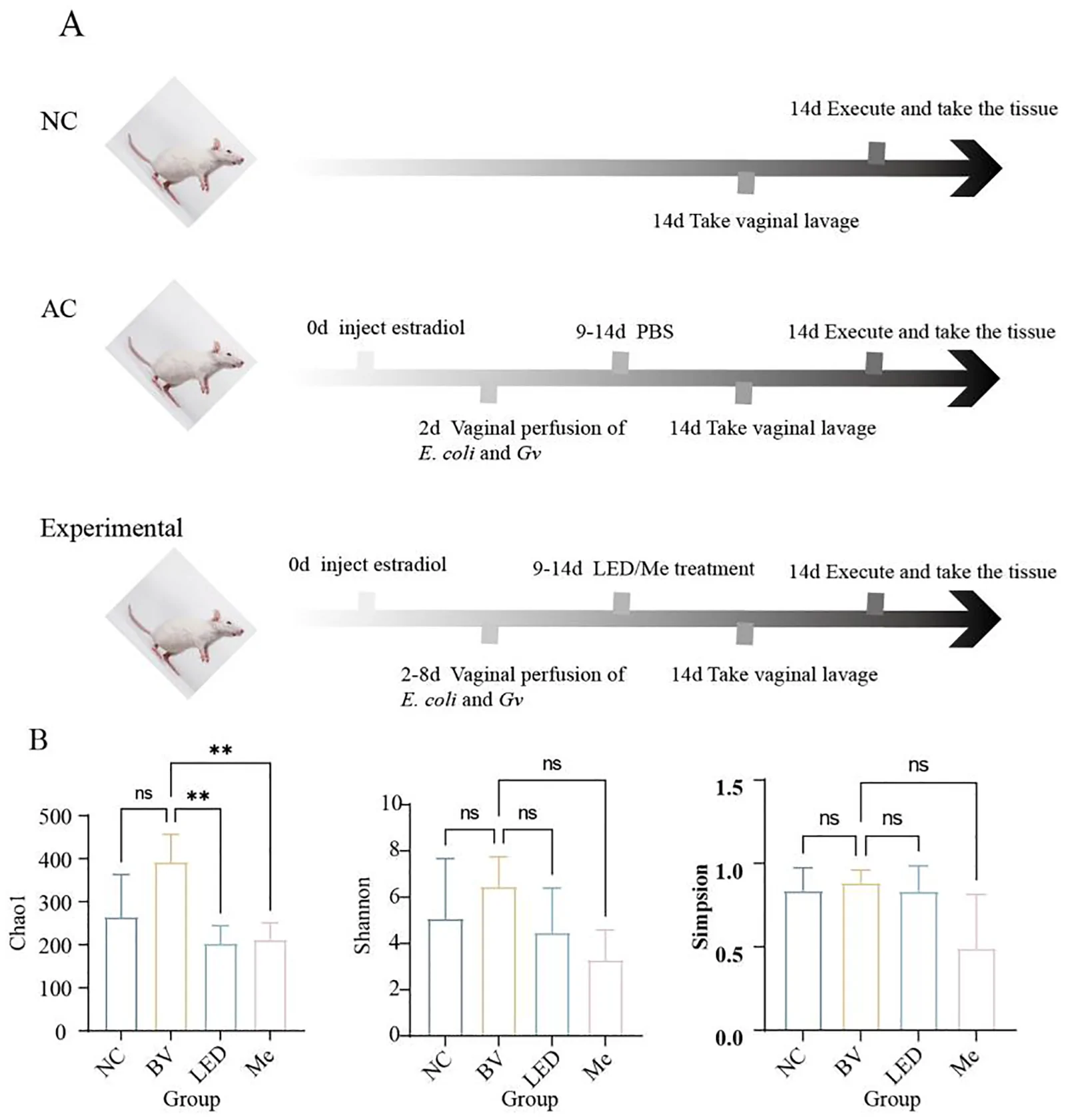
Alpha diversity analysis. **(A)** Animal experiment procedure **(B)** Statistical analysis of chao1 index, Shannon index, and simpsion index **P<0.01.

### 3.5 Effect of LED on vaginal tissue structure and inflammatory factor content in rats

Observation of uterine tissue revealed that rats in the model group (AC) exhibited localized congestion, whereas no significant congestion was observed in the normal control group (NC), LED-treated group (LED), or metronidazole-treated group (ME) (**Figure 5A**). HE staining results further revealed: The vaginal tissue structure in the normal control group (NC) was well-defined, with distinct layers of the mucosa, muscle layer, and lamina propria, and no inflammatory cell infiltration was observed.

**Figure 5:**
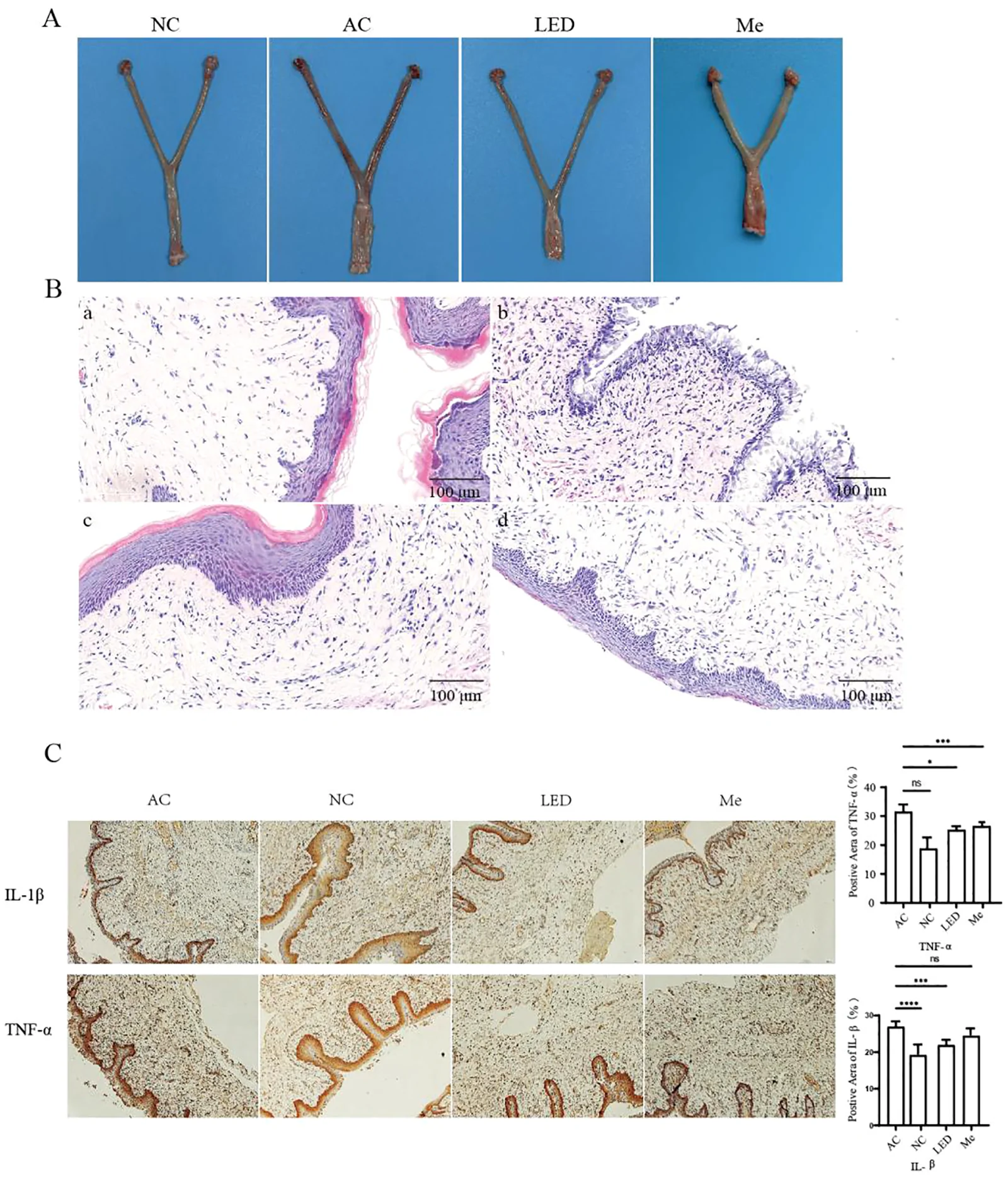
Effect of LED on rat vaginal tissue. **(A)** Uterine morphology; **(B)** HE staining of rat vaginal tissue, **(a)** NC, **(b)** AC, **(c)** LED, **(d)** Me. Scale bar: 100 μm; **(C)** IHC staining of rat vaginal tissue with TNF-α, IL-β. Magnification 20×. *P<0.05, ***P<0.001, ****P<0.0001, ns:not significant.

In the model group (AC), the vaginal mucosal layer structure was disorganized, tissue architecture was disrupted, cell arrangement was disordered, and significant inflammatory cell infiltration was present. The LED treatment group (LED) exhibited clear vaginal tissue architecture with distinct layers, showing only minimal inflammatory cell infiltration and no significant tissue damage. The metronidazole treatment group (ME) also demonstrated clear and intact structures in the vaginal mucosal layer, muscular layer, and lamina propria, with localized minimal inflammatory cell infiltration.

Immunohistochemical analysis revealed significantly higher expression levels of TNF-α and IL- 1β in vaginal tissues ofthe model group (AC) compared to the normal control group (NC) (P<0.05). Following LED and metronidazole treatment, the expression levels ofTNF-α and IL- 1β in vaginal tissues of both groups were significantly lower than those in the model group (AC) (P<0.05), indicating that both interventions effectively suppressed local inflammatory factor expression.

## 4 Discussion

The current issue of bacterial resistance has become a global health concern. The overuse and misuse of traditional antibiotics have accelerated the emergence of multidrug-resistant bacteria, making some common bacterial infections difficult to cure (Munita et al., 2016; Wang C-H. et al., 2020). Exploring diverse antimicrobial and therapeutic approaches has become particularly crucial.

The results of this study indicate that among LED light exposure at multiple wavelengths including 405 nm, 415 nm, and 430 nm, the 415 nm wavelength demonstrated the optimal *in vitro* antibacterial efficacy. The antibacterial action of LEDs primarily stems from their excitation of endogenous photosensitizers within bacteria to produce toxic substances such as reactive oxygen species (Angarano et al., 2020). Consequently, their bactericidal effect is closely related to the type and concentration of photosensitizers within the bacteria. Research indicates that zinc protoporphyrin IX is one of the common endogenous photosensitizers in various bacteria, exhibiting a significant absorption peak near 418 nm (Imtiaz et al., 2024). This spectral characteristic aligns with the observation in this study that 415 nm LED light delivers the most effective bactericidal results.

Notably, under identical experimental conditions, the LED demonstrated superior bactericidal efficacy against *Gv* compared to *E. coli*. Literature reports indicate that *E. coli* requires twice the light dose of Staphylococcus aureus to achieve comparable bactericidal rates, and LEDs typically exhibit higher antimicrobial activity against Gram-positive bacteria (eg, Staphylococcus aureus) than Gram-negative bacteria (eg.*E. coli*) *(*Bhavya and Hebbar, 2019).*Gv* is classically described as a Gram-variable or Gram-negative pleomorphic bacillus, and it exhibits heightened sensitivity to osmotic pressure and environmental fluctuations, with reduced viability in non-nutritive media like PBS. This susceptibility may partly explain its higher mortality rate in the experiment. In this study, the strain used was confirmed as Gram-positive via by Gram staining. Therefore, the observed stronger bactericidal effect of LED on *Gv* compared to *E. coli* aligns with the literature conclusion that LED exhibits greater efficacy against Gram-positive bacteria, further supporting the view that LED irradiation possesses a more pronounced antibacterial effect on *Gv* (Shvartsman et al., 2023).

Therefore, the observed stronger bactericidal effect of LED on *Gv* compared to *E. coli* aligns with the literature conclusion that “LED exhibits greater efficacy against ram-positive bacteria,” further supporting the view that LED irradiation possesses a more pronounced antibacterial effect on *Gv*.

Research findings indicate that significant increases in ROS levels were detected within both *Gv* and *E. coli* cells following LED irradiation, consistent with existing literature reports. LEDs primarily induce bacterial death by stimulating endogenous photosensitizers to generate ROS. Bacterial morphological observations revealed that LED treatment caused structural damage to the cell membranes and cell walls of both bacterial species, resulting in noticeable rupture. This phenomenon likely stems from the oxidative damage inflicted by ROS on the bacterial cell membrane system, aligning with conclusions from prior studies. However, other literature reports that even without ROS, LED irradiation can directly disrupt *E. coli* cell membrane structures through photophysical effects (Kim and Kang, 2021). Furthermore, this study found that CAT and SOD activities in *E. coli* were significantly reduced after LED exposure, This finding aligns with the results reported by KHAN S et al, suggesting that increased intracellular ROS levels may have exceeded the antioxidant capacity of bacterial enzymes, leading to structural damage consistent (Khan et al., 2019).This with literature conclusions identifying CAT as a potential target for antibacterial blue light (Guan et al., 2021). As an anaerobic bacterium, *Gv* may employ alternative antioxidant mechanisms. Regarding DNA damage, this study observed that LED treatment reduced total DNA content in both bacteria, consistent with some previous findings (Shi et al., 2022). However, other studies indicate that Pseudomonas aeruginosa genomic DNA showed no significant degradation under LED irradiation at bactericidal doses (Anjos et al., 2023). This study found that LEDs can cause damage at multiple sites in both *Gv* and *E. coli*, making it unlikely to induce bacterial resistance.

This study systematically evaluated the effects of 415 nm LED irradiation on VK2/E6E7 cells by measuring cell viability, intracellular ROS levels, and Live/Dead staining. Results indicate that under 415 nm LED irradiation at a dose of 90 J/cm², VK2/E6E7 cells exhibited enhanced metabolic activity. This increase in cell viability may stem from heat generated during LED exposure, elevating temperature and thereby boosting cellular metabolism. However, this study differs from some previous reports, such as literature indicating that 415 nm LEDs can reduce VK2/E6E7 cell viability (Wang T. et al., 2020). Further mathematical modeling analysis revealed that compared to 405 nm and 450 nm wavelengths, 415 nm LEDs exert the least impact on VK2/E6E7 cell activity. Furthermore, prior studies indicate that 405 nm and 450 nm LEDs require irradiation doses of 300 J/cm² and 1000 J/cm², respectively, to produce significant toxicity in lung epithelial cells (Bauer et al., 2021). The highest dose used in this study (90 J/cm²) is far below the aforementioned thresholds for inducing cellular toxicity, indicating that 415 nm LEDs possess high therapeutic safety when strictly controlled. Cellular ROS levels and Live/Dead staining results further confirm that 90 J/cm² of 415 nm LED irradiation did not cause significant cell death in VK2/E6E7 cells.

Notably, VK2/E6E7 cells contain significantly lower endogenous photosensitizer levels than bacteria (Wang et al., 2019). Consequently, 415 nm LED irradiation cannot efficiently excite intracellular photosensitizers to generate substantial ROS, thereby preventing cellular damage. In contrast, *Gv* and *E. coli* harbor higher intracellular photosensitizer concentrations, rendering them more sensitive to 415 nm LED irradiation.

In a rat model of bacterial vaginosis (BV), this study found that LED irradiation at a wavelength of 415 nm and a dose of 90 J/cm² did not significantly reduce the α-diversity index of the dominant vaginal microbiota in rats. This indicates that this irradiation condition did not markedly affect the overall species richness of the vaginal microbiome in rats. Histopathological analysis revealed that LED irradiation did not induce abnormal structural changes in vaginal tissues. Concurrently, IHC and inflammatory cytokine assays demonstrated significantly reduced expression levels of IL- 1β and TNF-α in vaginal tissues following LED intervention, suggesting that LED therapy can mitigate vaginal inflammation in rats. This finding aligns with literature reports indicating that LED light exposure neither alters normal histopathological structures in mouse tissues nor reduces expression levels of major inflammatory cytokines (Dai et al., 2013).

Furthermore, studies confirm that lactic acid bacteria do not require heme for growth, resulting in low porphyrin content within these microorganisms (Hadi et al., 2021). This strongly supports the minimal impact of antibacterial blue light on lactic acid bacteria during BV treatment, suggesting it effectively eliminates pathogenic bacteria while preserving vaginal microecological balance.

It should be noted that the current evidence is primarily derived from *in vitro* assays and a rat model with a limited sample size, and human clinical data are not yet available. Thus, although 415 nm LED irradiation showed potent antibacterial activity and favorable biosafety in experimental settings, the translational potential and risk profile of this approach in patients with vaginitis remain to be determined. Future work should involve well-powered, rigorously designed clinical trials to systematically validate the efficacy and safety of this phototherapeutic strategy. Prior to clinical adoption, several parameters need to be further refined, including optical power density, irradiation duration, treatment frequency, and cumulative dose. Moreover, individualized regimens adjusted according to the patient’s microbiological and inflammatory profile may offer a promising avenue for precision application.

## 5 Conclusion

This study provides experimental and theoretical support for the clinical treatment of vaginitis, but due to the lack of clinical studies, its safety and therapeutic effectiveness still need to be further evaluated. Therefore, the LED light power and light time used in the treatment in follow-up clinical studies can be further optimized and explored.

## Data Availability

The data presented in this study have been deposited in the NCBI repository under BioProject accession number PRJNA1518273.
